# Regional maldistribution of human resources of rehabilitation institutions in China Mainland based on spatial analysis

**DOI:** 10.3389/fpubh.2022.1028235

**Published:** 2022-11-08

**Authors:** Cheng Chen, Ting Chen, Ning Zhao, Siping Dong

**Affiliations:** ^1^School of Public Health, Wuhan University of Science and Technology, Wuhan, China; ^2^National Institute of Hospital Administration, National Health Commission, Beijing, China; ^3^Hospital Management Institute, Tsinghua University, Beijing, China; ^4^School of Political Science and Public Administration, Wuhan University, Wuhan, China; ^5^Health Research Institute, Fujian Medical University, Fuzhou, China

**Keywords:** rehabilitation institutions, rehabilitation professionals, human resource, allocation, disparities, spatial autocorrelation, spatial clustering

## Abstract

**Objective:**

With the demand for rehabilitation has been increasing faster for the aging of China's population, the equity of rehabilitation resource has aroused great concern. This study aimed to analyze the spatial distribution and evolution of the human resources of rehabilitation institutions and propose targeted countermeasures and suggestions to promote optimal allocation.

**Methods:**

A total of 31 provinces in China Mainland were identified and geocoded. The spatial weight matrix was introduced to analyze the spatial correlation. Spatial autocorrelation analysis and tests were used to analyze the spatial distribution and evolution characteristics of rehabilitation institutions' human resources in China from 2016 to 2019.

**Results:**

The average density of rehabilitation staff from 2016 to 2019 has been rising yearly (From 1.60 to 1.88). From 2018 to 2019, the proportion of rehabilitation professionals was higher than 75% in only 5 provinces, and no provinces met 75% from 2016 to 2017. Global Moran's I index from 2016 to 2019 showed no apparent aggregation phenomenon in the allocation of management personnel resources (*P* > 0.05). Three provinces in western China belonged to the Low-Low area and a province in northeastern China fitted to the Low–High area, with statistically significant differences. In addition, the changes in the spatial distribution and evolution trend of the human resources of rehabilitation institutions in different periods were affected by health policies.

**Conclusions:**

Although the overall spatial distribution gap of human resource allocation of rehabilitation institutions is shrinking, there are still internal structural defects and a maldistribution at the provincial level. It is necessary to improve the overall number of staff in rehabilitation institutions and to ameliorate the proportion of different types of staffing.

## Introduction

The demand for rehabilitation in China has been continuously growing due to the aging of China's population, the prevalence of chronic non-communicable diseases and unhealthy lifestyles (1). It is clearly stated in the outline of Healthy China 2030, which was published in 2016, to “Formulate and implement regulations on disability prevention and rehabilitation,” “Strengthen the construction of rehabilitation and care facilities for persons with disabilities,” and “Increase support for the construction of rehabilitation institutions in poor areas in Central and Western China.” The publication of the Health China 2030 implies that China has entered the ranks of the world's mainly rehabilitation countries.

Rehabilitation is an essential healthcare service for persons who are with a variety of medical issues. In addition to being the most efficient means to improve the physical state of people with disabilities, aid from rehabilitation institutions in staff is also the most effective way to increase their capacity for social adaptation. Rehabilitation for individuals with disabilities demonstrates a state's concern and attention for vulnerable groups like the disabled and the degree of society's overall development. Rehabilitation institutions and their human resources are the core elements that facilitate improved rehabilitation systems for the disabled. The availability of rehabilitation institutions, as well as their overall density, internal structure, and layout, is not only an important standard to measure the development level of rehabilitation services but also a reference basis for governments to propose targeted countermeasures and relevant policies (2).

Human resources in rehabilitation institutions still encounter challenges, including regional inequality and unbalanced development, although the state of rehabilitation technology in China has steadily risen. There is increasing interest in inequality in the context of the public health realm. Existing studies mainly focus on the following topics:

1. Specific rehabilitation issues, including comprehensive cardiovascular rehabilitation (3) and the conceptual evolution of neurorehabilitation (4);
2. Medical and health resource maldistribution, including the differences between urban and rural areas (5) and examinations of one specific province (6, 7);
3. Traditional research methods for the maldistribution or equity of health and medical resources, such as the Gini coefficient (8), Theil index (9), Lorenz curve (10), and Atkinson index (11).

Due to the lack of academic attention for rehabilitation human resource maldistribution, few studies concentrate on rehabilitation institutions' human resource maldistributions, especially in spatial research. The Theil index and health resource density index (HRDI), sensitive indices in evaluating the equality of resource allocation, have been used to explore the allocation of rehabilitation resources (12). However, when it comes to exploring the spatiotemporal interaction between different units (e.g., between provinces), it is difficult to effectively show the differences, regardless of whether using the Theil index or HRDI (13) and spatial aggregation analysis can overcome this deficiency. Meanwhile, most research objects are disabled people (14); little consideration is given to the needs of the whole population or potential target populations for rehabilitation resources.

We sought to answer increasingly complex questions rooted in social situations: What are the provincial trends, conditions, and maldistribution of human resources in China for rehabilitation? Are changes in rehabilitation human resource allocation affected by essential policies or important national conferences? In order to fully explore the trends and distribution of rehabilitation resources in various provinces of China, this study uses the provincial administrative regions as research units to express the spatial distribution and evolution characteristics of human resources of rehabilitation institutions at the provincial level from 2016 to 2019 visually through a geographic information system in order to encourage the steady development of rehabilitation in China and the rational use of resources, as well as offering suggestions for policymakers.

## Research design

### Variable selection

Rehabilitation professionals can be divided into three categories. They are Staff in Rehabilitation Institutions (SRI), Professionals (P), and Managerial Personnel (MP). In this study, we used “density” to analyze the distribution of human resources. The staff in rehabilitation institution density (SRID) represents the number of staff in rehabilitation institutions divided by population and multiplied by 10,000 (unit: persons per 10,000 population); professionals density (PD) represents the number of professionals divided by population and multiplied by 10,000 (unit: person per 10,000 population); and managerial personnel density (MPD), represents the number of managerial personnel divided by population and multiplied by 10,000 (unit: person per 10,000 population). The proportion of professionals (PP), which means the number of professionals divided by SRI (Unit: %), was also considered as an indicator for the guidance of the China Disabled Persons' Federation and the principle of post-structure proportion of professional and technical personnel in medical institutions (PRINCIPLE) formulated by Ministry of Health, PRC.

### Regional division

According to geographical location and gross domestic product per capita, provinces are grouped as belonging to three regions: the western, central, and eastern regions, representing the undeveloped, developing, and developed regions in China Mainland, respectively. The eastern region includes Beijing, Tianjin, Hebei, Liaoning, Shanghai, Jiangsu, Zhejiang, Fujian, Shandong, Guangdong, and Hainan. The central region includes Shanxi, Jilin, Heilongjiang, Anhui, Jiangxi, Henan, Hubei, and Hunan. The western area includes Inner Mongolia, Guangxi, Chongqing, Sichuan, Guizhou, Yunnan, Tibet, Shaanxi, Gansu, Qinghai, Ningxia, and Xinjiang.

### Data sources

The purpose of this study was to investigate the trends, spatial distribution, and evolution characteristics of human rehabilitation resources in China and to give evidence for policy decisions. To focus on the distribution of rehabilitation human resources in China under the background of Healthy China 2030, which was promulgated in 2016 and considers data availability, the analysis period started in 2016 and ended in 2019, the year of the latest available data.

We used the official statistics released by the China Statistical Yearbook on the Work for Persons with Disabilities (CSYWPDs) (15), which provided data on rehabilitation resources in 31 provinces, autonomies, and municipalities of China Mainland. The year-end resident population data items were from China Statistical Yearbook (2017–2020). The public welfare organizations that provide rehabilitation services to the disabled and other disabled drew their human resources from CSYWPDs. The 31 provincial areas were from the website of the National Bureau of Statistics. During the period covered by this study there has been no change in the number of provinces in China Mainland.

## Methods

Spatial autocorrelation was used to analyze the tendency of spatial clustering of human resources in rehabilitation.

### Spatial binary weight matrix

The spatial binary weight matrix (SBWM) was formed using GeoDa software (Version 1.16.0.12). We constructed our SBWM with the *Rook* criterion using the conventional method, holding that the weight is defined as 1 when two areas share a boundary but is valued at 0 otherwise (16, 17). However, Hainan Province has no common boundary with any other provinces because of its special geographical location. To facilitate analysis, we regarded Hainan as having a common boundary with Guangdong Province according to other scholars' research (18, 19). Table 1 shows the final result of spatial binary weight matrix.

**Table 1:** The final result of spatial binary weight matrix using *Rook* criterion.

**Number of neighbors**	**Frequency (names)**	**Number of neighbors**	**Frequency (names)**
1	1 (Hainan)	5	4 (Chongqing, Zhejiang, Guangdong, Guizhou)
2	4 (Beijing, Tianjin, Shanghai, Heilongjiang)	6	6 (Hubei, Hunan, Henan, Anhui, Jiangxi, Gansu)
3	5 (Liaoning, Jilin, Fujian, Ningxia, Xinjiang)	7	2 (Hebei, Sichuan)
4	7 (Shanxi, Tibet, Shandong, Jiangsu, Yunnan, Guangxi, Qinghai)	8	2 (Shaanxi, Inner Mongolia)

### Global spatial autocorrelation

Global spatial autocorrelation (GSA) is the spatial feature description of the characteristics related to resource (such as human resources) allocation in a whole specified area (20). Global Geary's C, Global Moran's I, and Global Getis-Ord G are commonly used indexes regarded as the statistics of GSA, of which Moran's I is the most widely used (21). This study used Global Moran's I index to explore the global spatial autocorrelation degree of human resources of rehabilitation institutions according to exploratory spatial data analysis. The formula of Moran's I is as follows:


$$Moran^{\prime }s\;I=\frac{n\sum_{i=1}^{n}\sum_{j=1}^{n}w_{ij}(x_{i}-\bar{x})(x_{j}-\bar{x})}{\sum_{i=1}^{n}\sum_{j=1}^{n}w_{ij}\sum_{i=1}^{n}{\left(x_{i}-\bar{x}\right)}^{2}}$$

In the above formula, x_*i*_ and x_*j*_ represent the observations of rehabilitation staff on space units *i* and *j*, respectively. $\bar{x}$ represents the rehabilitation staff density in the regional space of the study province. *w*_*ij*_ represents SBWM with the *Rook* criterion, and *n* represents the total number of provinces (in this study, *n* = 31) (22). For the difference in Global Moran's I index, the standardized statistical the Z-score test is generally used to reflect its significance level (23). The formula of the Z-score test is as follows (GeoDaSpace automatically runs Z-score test while calculating Moran's I index):


$$Z_{score}\;=\frac{I-E(I)}{\sqrt{VAR(I)}}$$

The value range of Moran's I is from −1 to 1. In this study, under the predetermined significance level (*p* < 0.05 in this study is significant), strong positive spatial correlation of human resources in rehabilitation institutions among provinces will appear if I is > 0 and closer to 1. If I is < 0 and closer to −1, it indicates that the negative spatial correlation between provinces is stronger. There is no spatial autocorrelation between provinces when I is 0 (24).

### Local spatial autocorrelation

The Global Moran's I index can describe the overall spatial autocorrelation of the distribution of human resources in China's rehabilitation institutions. However, there are different degrees of aggregation between different provinces and adjacent provinces at the local spatial level, which the Global Moran's I index cannot fully show. Local indicators of spatial association (LISA) often use the Local Moran's I index to capture important spatial patterns of clustering and dispersion at a local scale between local adjacent provinces and test the significance level (25). The principle is similar to the Global Moran's I index. In this study, the Local Moran's I index was selected to measure the spatial difference of human rehabilitation resources between adjacent provinces in China. The formula of the Local Moran's I is as follows:


$$Moran^{\prime }s\;I=\frac{\left(x_{i}-\bar{x})\sum_{j=1}^{n}w_{ij}(x_{j}-\bar{x}\right)}{S^{2}}\;,S^{2}=\frac{1}{n}\sum_{i=1}^{n}\;{\left(x_{j}-\bar{x}\right)}^{2}$$

In the above formula, x_*i*_ and x_*j*_ represent the observations of rehabilitation staff on space units *i* and *j*, respectively. $\bar{x}$ represents the rehabilitation staff density in the regional space of the study province. *w*_*ij*_ represents SBWM with the *Rook* criterion. $\left(x_{i}-\bar{x}\right)$ is the deviation between the average value and the observed value on the *i*th provincial unit, and *m* is the number of neighboring provinces of provincial unit *i* (for example, the m value of Hainan Province is 1). Furthermore, *n* represents the total number of provinces (in this study, *n* = 31). LSIA maps were drawn based on the Z-score significance level test under the predetermined significance level (*p* < 0.05 in this study is significant).

p40mm The Local Moran's I analysis results include four significant types that may be shown in LISA maps based on the size of the observed values: High–High type (HH), Low–Low type (LL), High–Low type (HL), Low–High type (LH), and one type with no significant difference (NG) (26). Each type represents different practical significance, indicating the spatial heterogeneity between adjacent provinces. A positive Moran's I value implies that the human resources in rehabilitation institutions were spatially clustered, and the spatial cluster types include high-high clusters (HH, high SRID, PD or MPD in a high SRID, PD or MPD neighborhood) and low-low clusters (LL, low SRID, PD or MPD in a low SRID, PD or MPD neighborhood); A negative Moran's I value implies that the human resources in rehabilitation institutions are staggered and evenly distributed (they are different from the surrounding zones) and include high-low outliers (HL, high SRID, PD or MPD in a low SRID, PD or MPD neighborhood) and low-high outliers (LH, low SRID, PD or MPD in a high SRID, PD or MPD neighborhood); Additionally, the NG type shows random distribution (27).

### Statistical analysis

IBM SPSS 26.0 was used for descriptive statistics to determine the number of human resources in rehabilitation institutions and the category composition of staff based on the number of people and service areas. Origin 2018 software was used to draw spatial Moran's I index curve maps.

## Results

### Regional distribution of rehabilitation human resources

#### The density of rehabilitation professionals

**Figure 1:**
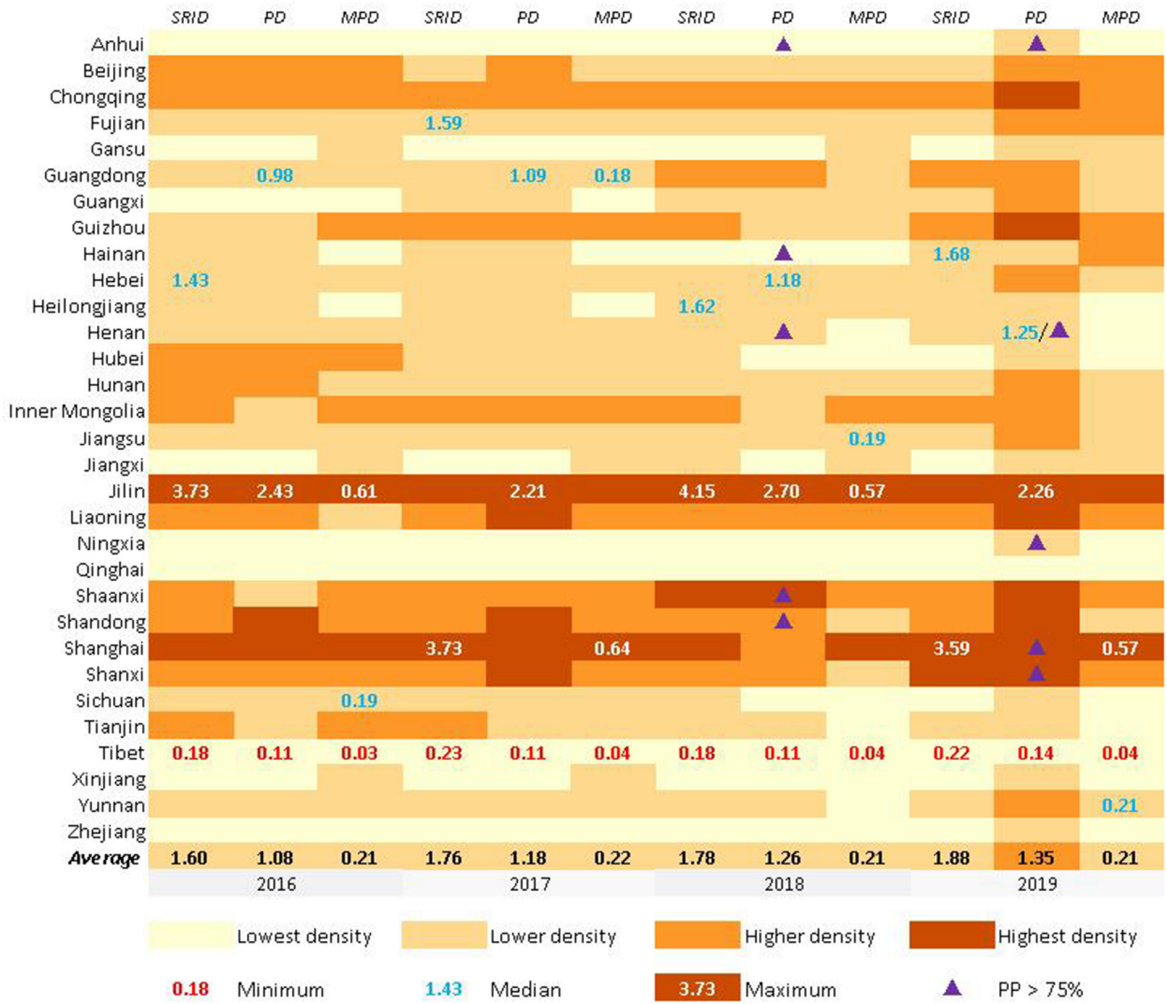
The density of rehabilitation staffs and from 2016 to 2019 in China.

cSRID, PD, and MPD from 2016 to 2019 are shown in Figure 1. Generally, the density of rehabilitation professionals showed a decreasing trend from the middle of the map to the east and west sides from 2016 to 2019. The density can be broken down into the “Lowest density” areas, the “Lower density” areas, the “Higher density” areas, and the “Highest density” areas, using “the Natural Breaks” method for four breakpoints.

SRID, PD, and MPD in Jilin all belonged to “Highest density ” areas while the three categories of staff in Qinghai and Tibet were all “Lowest density” areas from 2016 to 2019. The average SRID in China were 1.60, 1.76, 1.78, and 1.88 from 2016 to 2019, the average PD were 1.08, 1.18, 1.26, and 1.35 from 2016 to 2019, and the average MPD were 0.21, 0.22, 0.21, and 0.21 from 2016 to 2019. Other basic information can be seen in Figure 1. It is worth mentioning that SRID, PD, and MPD in Beijing and Shanghai were significantly higher than in their neighboring provinces (Beijing's neighboring provinces: Hebei and Tianjin. Shanghai's neighboring provinces: Zhejiang and Jiangsu).

#### The proportion of professionals in rehabilitation

The PRINCIPLE recommended that the proportion of professionals in rehabilitation institutions should reach 75%. From 2016 to 2017, no province met the PRINCIPLE's requirements. In 2018, five Chinese provinces' PPs were higher than 75%. They were Anhui (76.7%), Shandong (77.5%), Henan (78.6%), Hainan (76.1%), and Shaanxi (75.3%). In 2019, there were also five Chinese provinces' PPs higher than 75%. However, it was not totally the same provinces as in 2018, and they were Anhui (81.6%), Henan (78.5%), Ningxia (78.2%), Shandong (77.1%), and Shaanxi (76.7%), mainly non-coastal provinces. Among the 26 provinces below the recommended proportion, Jiangsu and Shanxi Provinces were close to reaching the standard of 75% (both 74.9%), and Shanghai (52.5%) had the lowest proportion (More details could be found in Figures 1, **5**–**8**).

### GSA of human resources in rehabilitation institutions

**Figure 2:**
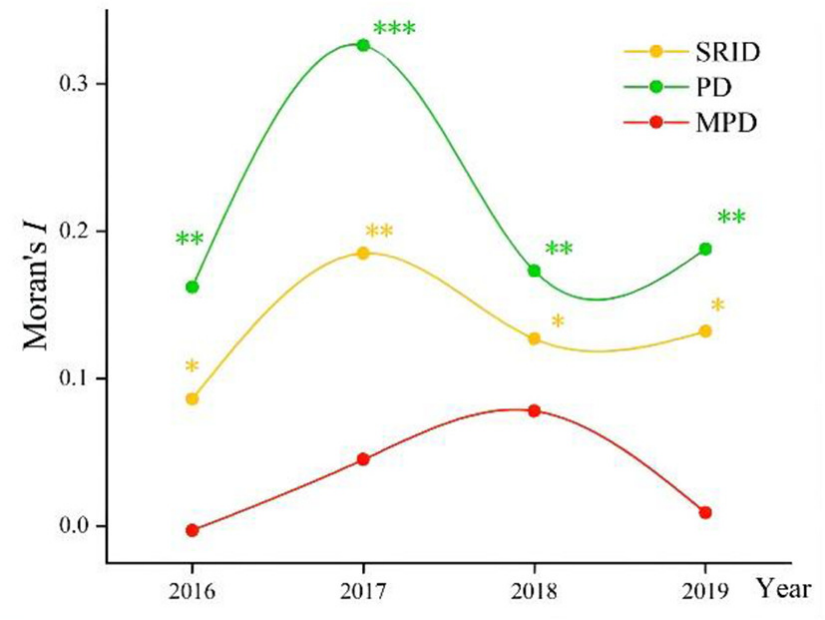
The changes in Global Moran's I index from 2016 to 2019 in China (allocated by population). *p < 0.05; **p < 0.01; ***p < 0.001.

**Figure 3:**
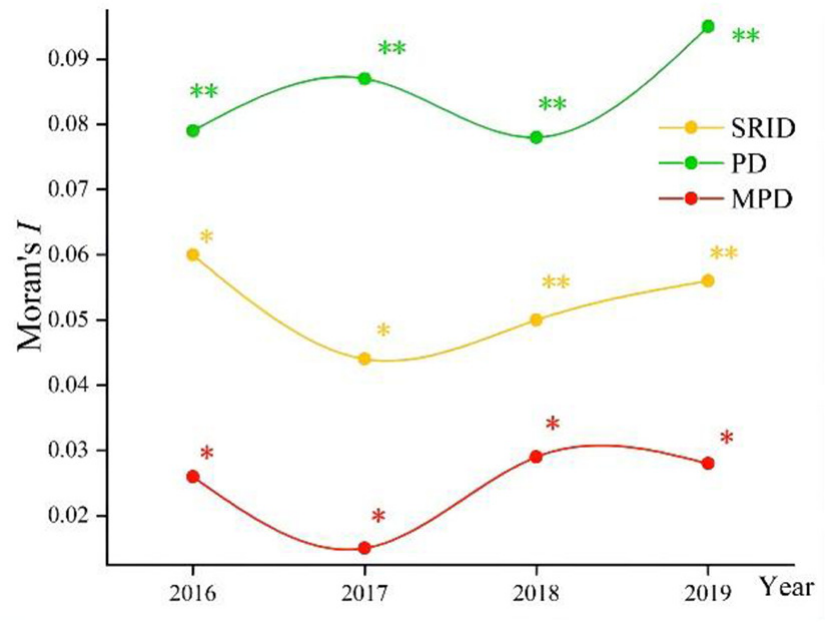
The changes in Global Moran's I index from 2016 to 2019 in China (allocated by regional area). *p < 0.05; **p < 0.01; ***p < 0.001.

The changes in Global Moran's I index from 2016 to 2019 are shown in Figures 2, 3 (with 999 randomized replacement Z-score tests). The results clearly illustrated differences in the aggregation of rehabilitation staff between allocation by population density and provincial area. On the whole, the difference in human resources in rehabilitation institutions among provinces continued to fluctuate, and the degree of spatial clustering did not continue to decline or rise. However, regardless of the absolute value or fluctuation range of the index, the spatial autocorrelation of human resources in rehabilitation institutions allocated by population showed a higher aggregation than that allocated by regional area. Thus, human resources in rehabilitation institutions allocated by regional area were fairer than those allocated by population. MP allocated by population had no statistical significance in the global autocorrelation test (*p* > 0.05), which means there was no apparent aggregation phenomenon in the allocation of MP resources.

### LSA of human resources in rehabilitation institutions

The LISA significance test showed the provincial spatial unit attributes of rehabilitation personnel and their interaction with adjacent provinces in China from 2016 to 2019.

**Figure 4:**
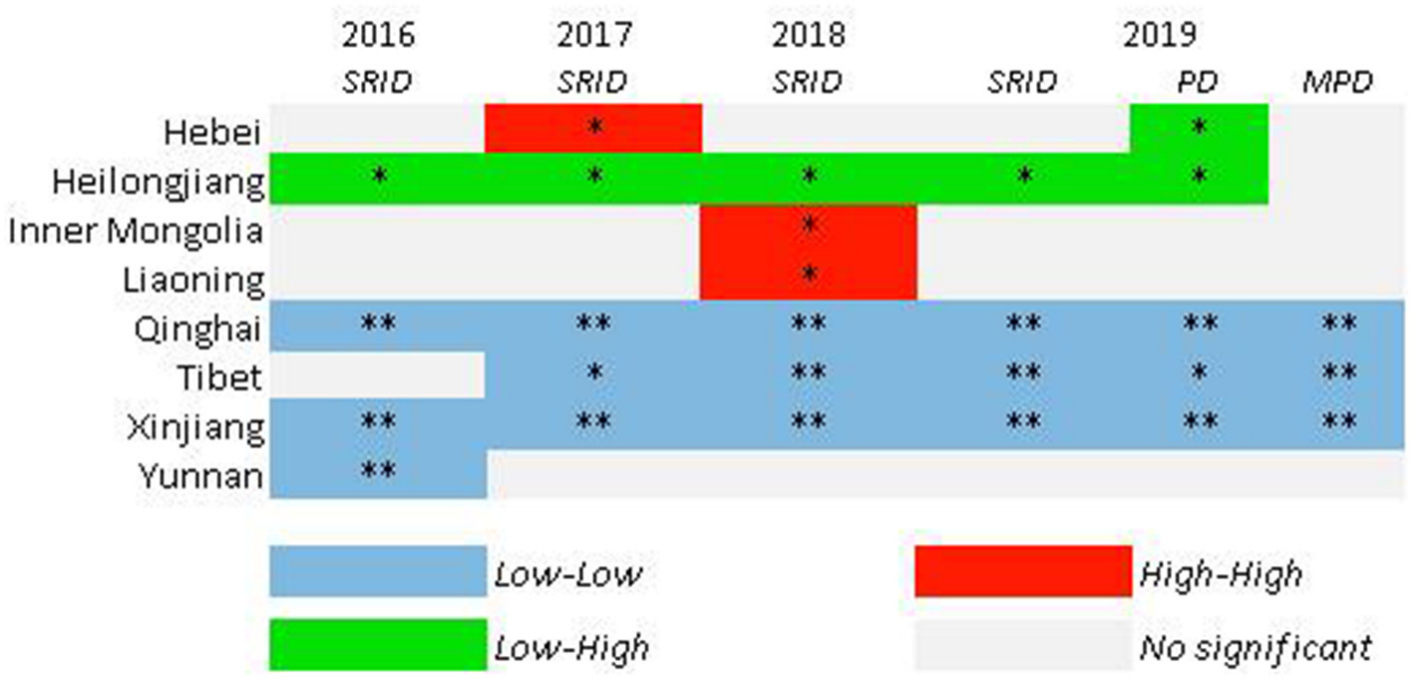
LISA maps for SRID from 2016 to 2019; PD and MPD in 2019. *p < 0.05; **p <0.01; ***p < 0.001. Z-score test, 999 randomized replacement. Other provinces not shown means that all the test indicators were not significant.

It could be clearly seen that the distribution of SRI resources in China has been relatively consistent as a whole in the past 4 years (Figure 4), although there was a slight fluctuation every year. There was no noticeable fluctuation in LSA. The overall distribution situation was the LL type in northwest and west China, LH type in northeast China (Heilongjiang), and occasionally the HH type.

The LISA test results also showed a difference between SRID, PD, and MPD in the same year. Figure 4 shows that SRID, PD, and MPD in Tibet, Xinjiang, and Qinghai belonged to the Low–Low type, while SRID and PD in Heilongjiang were the Low–High type, and there was no apparent aggregation for MPD. Hebei belonged to the Low–High type in terms of PD.

### Structural changes in rehabilitation staff in rehabilitation institutions

**Figure 5:**
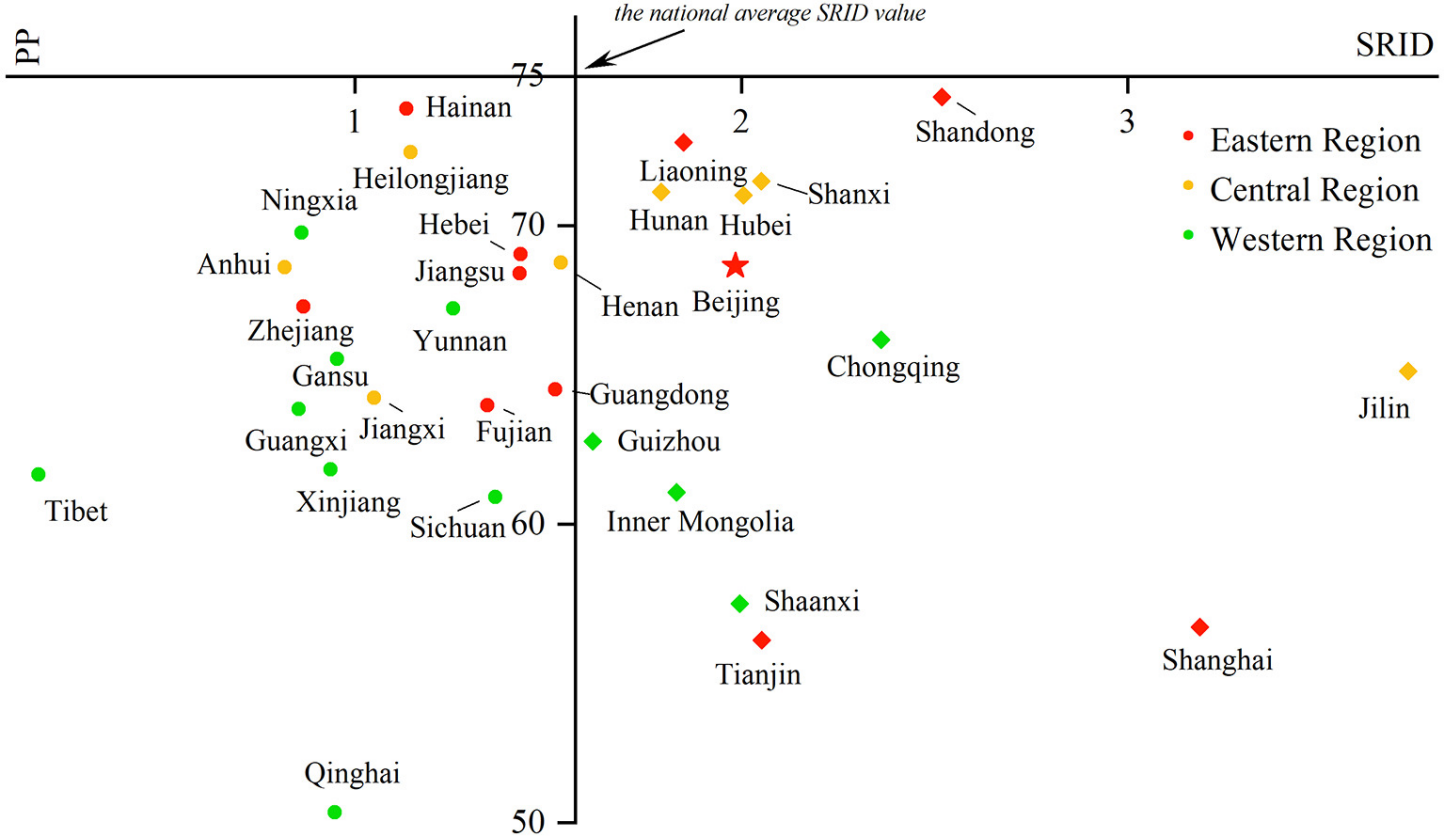
Scatter plot with four quadrants for the proportion of professionals in 2016.

**Figure 6:**
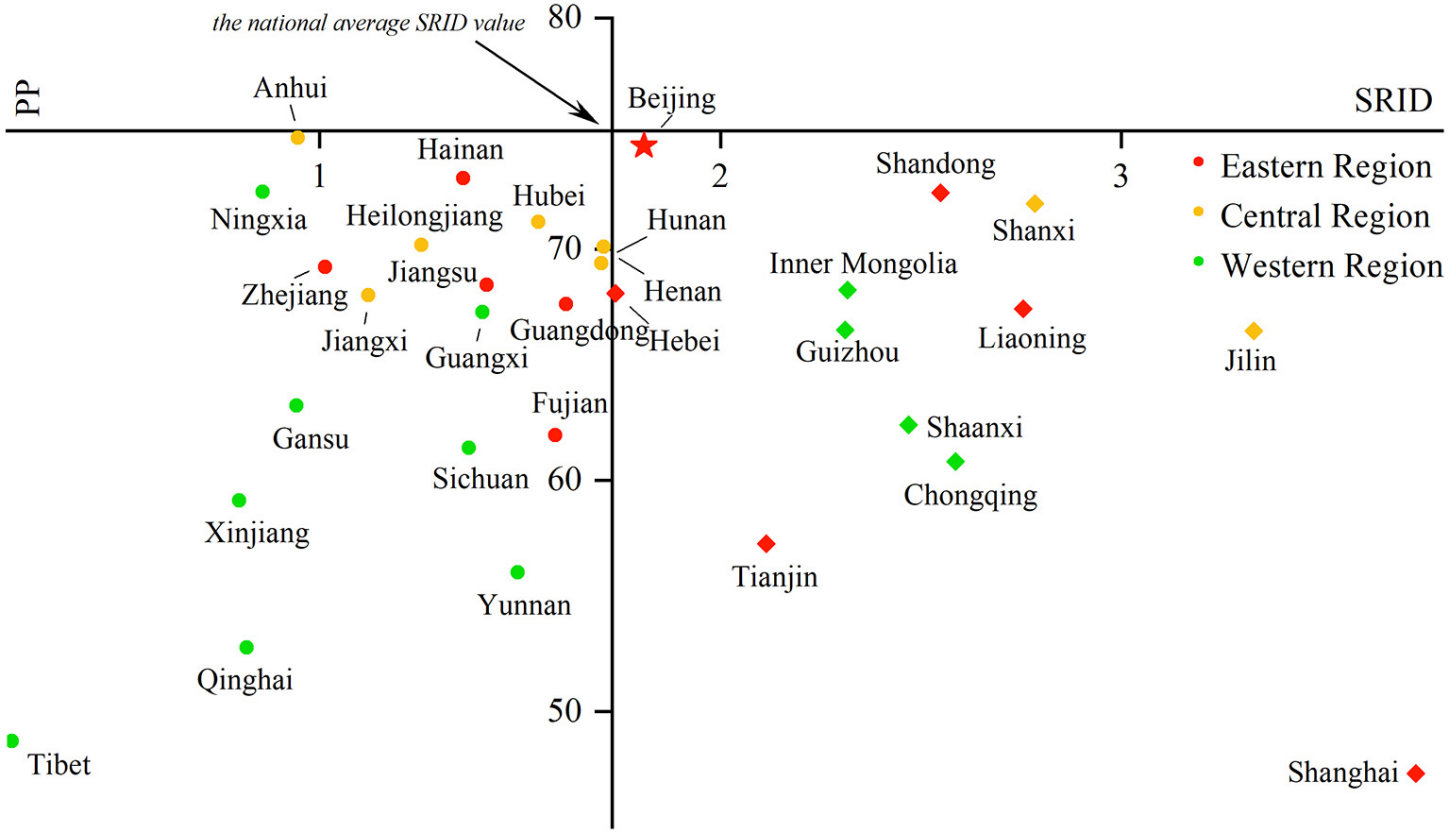
Scatter plot with four quadrants for the proportion of professionals in 2017.

**Figure 7:**
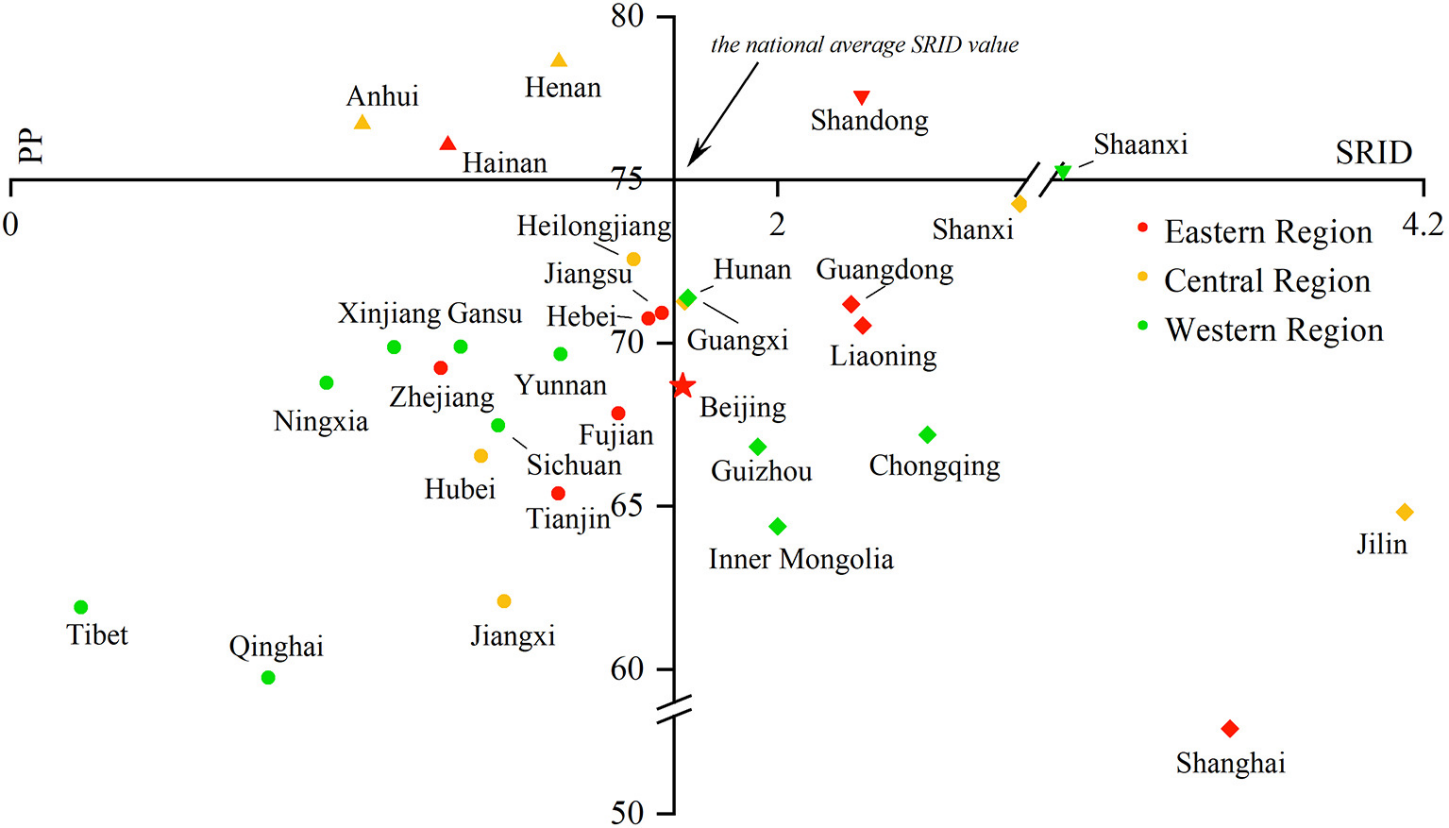
Scatter plot with four quadrants for the proportion of professionals in 2018.

**Figure 8:**
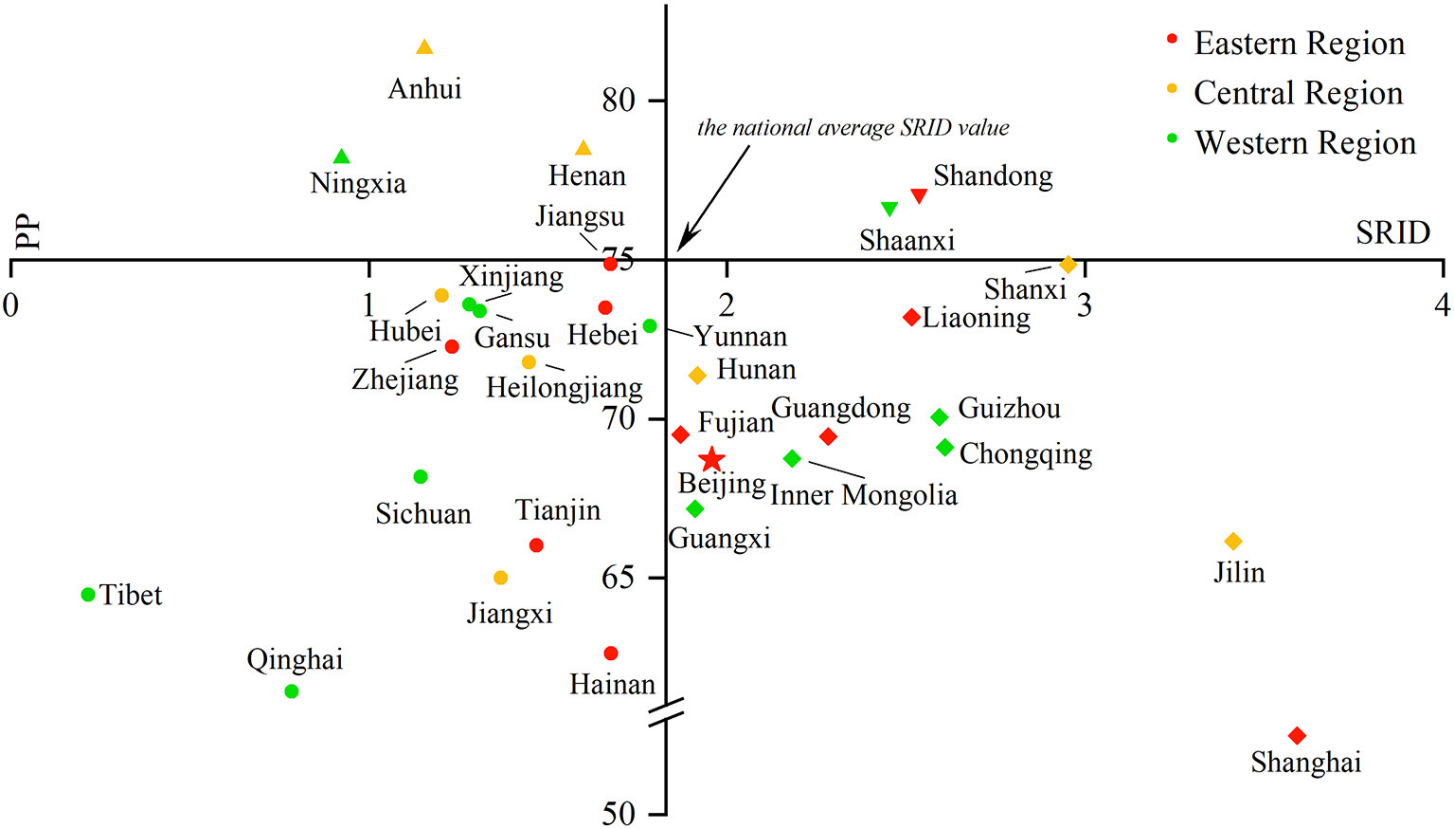
Scatter plot with four quadrants for the proportion of professionals in 2019.

Comparing the SRID and PP in rehabilitation institutions from 2016 to 2019 can reflect the changes in the internal structure of rehabilitation staff in the past four years to a certain extent. With the SRID as the horizontal axis and PP as the vertical axis, the scatter plot with four quadrants was drawn using Origin 2018 software. The four quadrants have the following meanings:

In the first quadrant, the SRID of the province is higher than the national average, and the PP is higher than the PRINCIPLE's recommendation. For the second quadrant, the PP meets the suggestion of the PRINCIPLE, but the SRID is lower than the national average. The SRID is lower than the national average, and the PP is lower than the PRINCIPLE of advice in the third quadrant. As for the fourth quadrant, the SRID is higher than the national average, but the PP is lower than the requirements in the PRINCIPLE. The scatter plot is displayed in Figures 5–8.

From 2016 to 2017, there was no province in the first and second quadrants, as they were mainly scattered in the third and fourth quadrants and gradually “migrated” from the third quadrant to other quadrants over time. That is, the PP in an increasing number of provinces has begun to meet the PRINCIPLE, or the SRID has been reaching the national average. From 2018 to 2019, Shandong and Shaanxi, which were distributed in the first quadrant, took the lead in meeting the two standards. The western region was mostly concentrated in the third quadrant, which means these provinces failed to meet the two indicators. The eastern and central regions were mostly concentrated near the two axes, which suggested that the staffing of these provinces was close to the national average. Over the past 4 years, the average value of the SRID in China has been gradually increasing (the vertical axis has been moving to the right). It should be pointed out that the position of Tibet and Shanghai in the scatter plot did not change. When the PP was also low in these two provinces, the SRID showed the opposite phenomenon of one being high (Shanghai) and one being low (Tibet).

## Discussion

This was a nationwide study that comprehensively evaluated the trend of allocation of human resources in China's rehabilitation institutions by longitudinal and horizontal comparison. Rehabilitation is a fundamental health service for people with a variety of health conditions, and it primarily focuses on improving their ability to function well in society and on reducing the impact of disability (28).

### Human resource maldistribution and staff structure

From the perspective of the whole country, there were certainly regional differences between provinces in 2019, regardless of whether SRID, PD, or MPD was examined, with a massive gap of approximately 16 times between the highest and lowest provinces. As far as the provincial level is concerned, Tibet was the worst, as its SRID was only 0.22, which suggests that local residents are not able to obtain rehabilitation resources on time.

In the meanwhile, the internal staff structure of rehabilitation institutions also needs to be improved. According to the examination of this study, from 2016 to 2017, no province implemented the PRINCIPLE's recommendation for the PP. Although this phenomenon has been ameliorated in the past 2 years, the number of qualified provinces remained low (no more than five provinces, 17%), which suggests that there may be some defects in the staff structure in all provinces, for example, the number of SRI being insufficient or there being too many MPs and other personnel. Determining how to increase the PP to improve maldistribution appropriately is a task that needs further attention in some provinces.

Therefore, relevant solutions and schemes should be considered at the national level to improve the PP in rehabilitation institutions in different provinces. For policy formulation and implementation, we should focus more attention on the following points:

(1) While the spatial gap of human resources in rehabilitation institutions has been gradually narrowing, the SRID in western China has maintained a low level, which may indicate the lack of rehabilitation resources and severe brain drain in western China.

(2) Those provinces where the PP does not meet the principles should pay attention to the adjustment of personnel internal structure, balance workload and efficiency, give consideration to quantity and structure, and further optimize the human resource allocation of rehabilitation institutions while expanding the number of SRI.

### Effect of health policies on spatial clustering

The trend of the Global Moran's I index reflected that although the difference in human resources allocated by the regional area of rehabilitation institutions in China was small, the spatial autocorrelation of human resources allocated by populations still needed to improve. The mention of “Accelerating talented professionals in rehabilitation” and other pertinent topics in the National People's Congress and the Chinese People's Political Consultative Conference in 2017, as well as the regulations on disability prevention and rehabilitation promulgated by the State Council, may be responsible for the Global Moran's I index's decline and stabilization after it peaked in 2017. The impact of major conferences or policies in the field of health on the spatial autocorrelation of human resource development in rehabilitation institutions in China may be a process of fluctuation and narrowing. The specific impact changes need further studies.

Therefore, the effectiveness of health policies, especially those related to the construction of rehabilitation institutions and the training of rehabilitation specialists, should be paid more attention to. The continuous role of such policies will affect the spatial distribution of staff in rehabilitation institutions.

### General trends and regional differences

Due to the external interference factors, such as the social economy and natural environment, there will be varying degrees of spatial heterogeneity when a large area is selected for research (29, 30). Based on this theory, this study combined global spatial autocorrelation analysis with local spatial autocorrelation analysis to evaluate the human resource allocation of rehabilitation institutions among provinces. Local Moran's I index showed that there was spatial autocorrelation in some provinces or regions, and the east and west regions showed somewhat opposite trends to each other, which were mainly reflected in the fact that Low–High aggregation continued in the northeast (especially Heilongjiang), whereas the west region always showed Low–Low aggregation. The main reasons may include the following:

(1) The human resources of rehabilitation institutions had a certain spatial spillover effect. Under the interaction of adjacent provinces, the investment in human resources of this province was affected by the personnel flow of neighboring provinces to a certain extent, and this led to a continuous “depressions” effect in Heilongjiang from 2016 to 2019 (31). This could be explained by the fact that under the influence of the social economy and natural environment, rehabilitation professionals in Heilongjiang may prefer neighboring provinces, such as Inner Mongolia and Jilin, for employment and resettlement, resulting in the continuous brain drain in Heilongjiang.
(2) Due to its vast territory, sparsely populated area, and relatively backward economic development compared with other regions, it is difficult for the provinces in western China to gain adequate attraction for rehabilitation professionals. On this basis, the investment and attention of local governments in the rehabilitation industry will decrease, which will result in further specialist scarcity and ultimately form a destructive cycle. This explains why those provinces in western China mostly showed Low–Low aggregation.

### A reasonable “SRID” index is needed

Compared with other medical and health institutions and the Centers for Disease Control and Prevention in China, which have clear provisions on their staffing ratio, there is a lack of regulations regarding the number and proportion of staff in rehabilitation institutions in China. The lack of regulations may lead to some errors when studying the allocation of rehabilitation staff in various provinces. Establishing a reasonable SRID index is a key and effective step to improve the maldistribution of human resources of rehabilitation institutions in China.

### Various results between different target groups

The result of spatial clustering in this study was quite different from other scholars' research results of human resources allocation (human resources allocated by population were fairer than allocated by regional area), which might be caused by different research objects (32). Previous studies mostly focused on the disabled, only considered the needs and access of the disabled to rehabilitation resources, and ignored the needs of the whole population or potential population for rehabilitation resources. This study produced different results due to the expansion of the scope of the target population.

The unique study results also suggest that systematic and multi-factor comprehensive consideration should be taken into account while making a research design processing and in the analysis of resource allocation so as to reasonably define the scope of the target population and promote the formulation of reasonable and effective policies.

### Limitations

The result of LISA showed too many NG types in most provinces. One of the possible reason for is that the extremely significant aggregation in very few regions/provinces obviously covers up the aggregation in other regions. More microscopic research needs to study further like central region in China.

This study focused more on the gaps between different provinces. Actually, the gaps within provinces also exist, and the degree of economic development in provinces may show different maldistribution. On the other hand, the duration of the study started from 2016 to 2019 due to the different statistical indicators in the CSYWPDs before 2016 and the pandemic of COVID-19 in 2020, which led to a short study period. Future research should explore the gaps within provinces and a long-term analysis on regional distribution of human resources of rehabilitation institutions.

## Conclusions

Spatial statistical analysis can intuitively and continuously analyze agglomeration and changes caused by certain factors at different time nodes. There are still several inequities regarding allocating human resources in rehabilitation institutions in China. The most prominent one is that PP was relatively low, and rehabilitation specialists were strongly demanded in most provinces. In order to ensure that the rehabilitation work for the disabled in China is in line with international standards and develops smoothly, the following recommendations were put forward for reference : (1) To effectively analyze the main rehabilitation needs of the disabled in China, a long-term plan for the training, education, certification, and use of rehabilitation professionals should be formulated; (2) A reasonable “SRID” index of rehabilitation institutions that meets the specific conditions of each province, which could provide guarantees and references to evaluate the overall situation and formulate relevant health policies, should be developed; (3) A reasonable target population range with resource demands for improving the effectiveness of policy implementation should be considered.

## Data availability statement

Publicly available datasets were analyzed in this study. This data can be found at: http://www.zgtjcbs.com; http://www.stats.gov.cn/tjsj/ndsj/2019/indexch.htm.

## Author contributions

CC contributed to material preparation, data analysis, and the first draft of the manuscript. TC contributed to data collection, the conception of the study, and modification opinions. NZ was responsible for polishing the article. SD contributed significantly to analysis and manuscript preparation, helped perform the analysis with constructive discussions, and polish the article to make it more readable. CC, TC, NZ, and SD contributed to the study's conception and design and commented on previous versions of the manuscript. All authors read and approved the final manuscript.

## Funding

This research was funded by the National Key Research and Development Plan of China (Grant No. 2020YFC2006000). The funders had no role in the study design, data collection and analysis, decision to publish, and preparation of the manuscript.

## Conflict of interest

The authors declare that the research was conducted in the absence of any commercial or financial relationships that could be construed as a potential conflict of interest.

## Publisher's note

All claims expressed in this article are solely those of the authors and do not necessarily represent those of their affiliated organizations, or those of the publisher, the editors and the reviewers. Any product that may be evaluated in this article, or claim that may be made by its manufacturer, is not guaranteed or endorsed by the publisher.
